# *GhGLK1* a Key Candidate Gene From GARP Family Enhances Cold and Drought Stress Tolerance in Cotton

**DOI:** 10.3389/fpls.2021.759312

**Published:** 2021-12-16

**Authors:** Jiangna Liu, Teame Gereziher Mehari, Yanchao Xu, Muhammad Jawad Umer, Yuqing Hou, Yuhong Wang, Renhai Peng, Kunbo Wang, Xiaoyan Cai, Zhongli Zhou, Fang Liu

**Affiliations:** ^1^State Key Laboratory of Cotton Biology, Institute of Cotton Research, Chinese Academy of Agricultural Sciences, Anyang, China; ^2^Anyang Institute of Technology, Anyang, China; ^3^School of Agricultural Sciences, Zhengzhou University, Zhengzhou, China

**Keywords:** cotton, cold stress, drought stress, *GhGLK1*, *Arabidopsis thaliana*

## Abstract

Drought and low-temperature stresses are the most prominent abiotic stresses affecting cotton. Wild cotton being exposed to harsh environments has more potential to cope with both biotic and abiotic stresses. Exploiting wild cotton material to induce resistant germplasm would be of greater interest. The candidate gene was identified in the BC2F2 population among *Gossypium tomentosum* and *Gossypium hirsutum* as wild male donor parent noted for its drought tolerance and the recurrent parent and a high yielding but drought susceptible species by genotyping by sequencing (GBS) mapping. Golden2-like (GLK) gene, which belongs to the GARP family, is a kind of plant-specific transcription factor (TF) that was silenced by virus-induced gene silencing (VIGS). Silencing of GhGLK1 in cotton results in more damage to plants under drought and cold stress as compared with wild type (WT). The overexpression of GhGLK1 in *Arabidopsis thaliana* showed that the overexpressing plants showed more adaptability than the WT after drought and cold treatments. The results of trypan blue and 3,3′-diaminobenzidine (DAB) staining showed that after drought and cold treatment, the leaf damage in GhGLK1 overexpressed plants was less as compared with the WT, and the ion permeability was also lower. This study suggested that the GhGLK1 gene may be involved in the regulation of drought and cold stress response in cotton. Our current research findings add significantly to the existing knowledge of cold and drought stress tolerance in cotton.

## Introduction

Plants are exposed to a variety of harsh environmental stresses (i.e., cold, heat, and drought), affecting the photosynthetic rate and normal physiological metabolisms, thus leading to plant death (Morales et al., 2020). Drought has had a significant impact on crop yields. Drought-induced food output losses have cost the world $30 billion over the last decade, according to the World Food and Agriculture Organization. To make the best use of water resources, it is critical to investigate the mechanisms of plant response to drought stress (Zhang et al., 2021). Cold stress can adversely affect plant growth and development, including inhibiting seed germination, slowing plant growth, hindering reproduction, reducing crop yields and quality, and limiting the geographical distribution of species (Körner, 2016). As a result, many crops, such as rice, maize, tomatoes, soybeans, and cultivated cotton, cannot adapt to low temperatures and can only grow in tropical or subtropical regions (Gong et al., 2020).

Osmotic regulation plays a significant part in the resistance of crops to numerous non-biological stresses, and plants can maintain the osmotic pressure within cells by increasing solutes in the cells, preventing water dispersion (Evelin et al., 2019). Permeable substances can also be used as solvents to participate in biochemical reactions or to bind to proteins, thereby reinforcing the structure of proteins. Free proline functions as an osmotic regulator, and research has revealed that when rice is stressed by drought, it accumulates high amounts of proline (Rodrigues et al., 2019). Antioxidant regulation mechanism is another important regulatory mechanism to cope with drought stress in plants. Under normal growth conditions, the active oxygen in the body is in equilibrium. Drought stress induces the production of reactive oxygen in plant chlorophyll and mitochondria, which destroys cell integrity and damages plants, eventually producing malondialdehyde (MDA), the final product of membrane peroxides (Stepien and Klobus, 2005). The reactive oxygen system produces substances such as superoxide dismutase (SOD), peroxidase (POD), catalase (CAT), glutathione peroxidase (GPX), ascorbic acid (ASA), and glutathione, which are accompanied by an increase in reactive oxygen, to limit and regulate the damage of reactive oxygen to plants, but also as a signal molecule to activate the plant body to respond to the external adverse environment (Asada, 2006; Ahmed et al., 2013).

Under cold stress, crops resist the effects of low temperature by regulating the composition of the membrane system, activating reactive oxygen removal system, promoting cold stress response to gene expression, and regulating hormone content and osmotic regulatory substances (Miura and Furumoto, 2013). Under normal growth conditions, the low level of reactive oxygen of the body will not cause damage to plants, and with the production of excess reactive oxygen, plants constantly remove the high concentration (Sharma et al., 2012). However, free radical production increases under cold stress, and to counteract the start of protective enzyme systems in these free radical plants, rapid synthesis of SOD, glutathione reductase (GR), ascorbic acid peroxidase (APX), POD, and so on will reduce the damage of reactive oxygen to plants (Hasanuzzaman et al., 2020). Cold stress response genes, such as RD29A, COR15, KIN1, and COR47, have been reported to play a vital part in defending plants against cold stress (Lee and Lee, 2000). In addition, defensive proteins, such as antifreeze proteins, dehydrated proteins, and heat proteins, encoded by cold stress response genes, also play a key role in enhancing cold stress tolerance in crops. Hormonal contents in plants are also related to their cooling resistance, and findings have revealed that abscisic acid (ABA) can regulate plant response to low temperatures, and the addition of ABA can induce the expression of cold response genes (Fan et al., 2002).

In Xinjiang, which is the main cotton-producing region in China, extreme environments such as low temperature and drought are major problems and challenges in cotton production (Li et al., 2020). Cotton is a warm crop, more sensitive to the low-temperature environment. Improving tolerance of cotton to low temperature, on the one hand, can be through the seedling period of low-temperature exercise to tolerate the adverse effects of low temperature. This phenomenon is called cold domestication, which Guy et al. (1985) first proposed in 1985, and many plants increase their cold resistance after a period of non-freezing temperatures. On the other hand, the use of existing seed resources, mining cotton cold-resistant genes, to cultivate more cold-resistant cotton varieties, is very crucial (Shi et al., 2018).

The Golden2-like (GLK) gene, first found in corn belonging to the GARP family (Murmu et al., 2014), is a plant-specific transcription factor (TF) that plays a key role in regulating plant growth, disease resistance, hormone signal transduction, and maintenance of circadian rhythms (Riechmann et al., 2000). In anthropomorphic mustard, the GARP family has gene members to GLK, as well as the Type B arabidopsis response regulators (ARR) protein gene that encodes the N-end domain. The homologous gene of GLKs has also been found in rice, peanuts, tomatoes, and chili peppers (Powell et al., 2012; Safi et al., 2017). The GLK gene is an activator for chlorophyll biosynthesis, photo collection, and electron transmission of nuclear photosynthesis genes that act in a cell-autonomous manner to manage and sustain photosynthesis within a single cell (Waters et al., 2008). It can also play a role in photosynthesis optimization by coordinating responses to flexible environments and endogenous signs. Previous studies have shown that the GLK1/2 can regulate chlorophyll synthesis, chlorophyll development, and other processes in sugar cane, rice, corn, and *Arabidopsis* (Waters et al., 2008; Chen et al., 2016). GLK1/2 double mutation experience makes the shape of rice change, double mutant plant in relative to the wild type (WT) of light color (Fitter et al., 2002). The experimental findings of Liu et al. (2018) show that the transformation of the peanut GLK gene into *Arabidopsis* has improved its drought tolerance by affecting the morphological development and photosynthesis of *Arabidopsis*. The GLK gene can also regulate multiple ABA response genes, such as WRKY40 (Ahmad et al., 2019).

The primary task of cotton breeding is cultivating the higher quality of drought and cold tolerant varieties and exploring the drought and cold tolerance mechanism of cotton. Therefore, it is of great significance to excavate the potential genes from wild cotton and transfer them to cultivated varieties, which is of great implication to the improvement of cotton varieties. In this study, in the GLK gene, “GhGLK1” was identified as a key gene by quantitative trait loci (QTL) placement of drought-resistant characteristics in the terrestrial cotton of the BC_2_F_2_ population, and its upregulated expression was confirmed by real-time quantitative PCR (RT-qPCR) to be a candidate gene for enhancing drought resistance in cotton. This study explores the function of the GhGLK1 gene under cold and drought stress through virus-induced gene silencing (VIGS) in cotton and overexpression in *Arabidopsis thaliana*.

## Materials and Methods

### Experimental Materials and Treatments

The candidate gene “GhGLK1” was identified in the BC_2_F_2_ population among the wild male donor parent, i.e., *Gossypium Tomentosum*, which is noted for its drought tolerance, and the recurrent parent, i.e., *Gossypium hirsutum*, a high yielding but drought susceptible species *via* genotype by sequencing (Magwanga et al., 2018). *A. thaliana* (Col-0) was screened from T0 to T3. The sterilized seeds were grown on a 1/2 murashige and skoog (MS) medium and then allowed to grow in suitable 22°C (16 h light/8 h dark) conditions. Then, the plants were transplanted into a nutrient mixture of nutrient soil and vermiculite with a 1:1 ratio. For cold stress treatment, after 3–4 weeks of transplantation, we selected the consistent WT and overexpression T3 generation plants of the *Arabidopsis* and placed them in a refrigerator at 4°C. After 7 days of cold treatment, the survival rates of plants were recorded. To simulate drought, 16% PEG6000 solution was applied, and germination rate and root length were determined using the 1/2 MS medium containing mannitol. Mannitol concentrations were set to 100, 200, and 300 mM in three concentration gradients, with the optimum concentration of 200 mM mannitol selected after pretesting. Each plastic canopy contains five *Arabidopsis* seedlings, each set of 12 boxes (Lu et al., 2019).

Marie Galante-85, a wild species of terrestrial cotton, was used in the experiment, and the carrier was TRV2. Seeds were soaked, the filter paper method was used for sowing, and 4 days after sprouting were transplanted into a hydroponic setup filled with Hoagland nutrients. Greenhouse conditions were 28°C during the day, 25°C at night with 16 h/8 h light/dark cycle, and relative humidity of 60–70%. The plants were moved to a 4°C growth chamber for the cold stress treatment, and then samples were taken at 0, 3, 6, 12, and 24 h treatment time. Immediately after sampling, the leaf samples are collected in liquid nitrogen and stored at a −80°C refrigerator until RNA was extracted. The RNAprep plant kit, which is supplied by Tiangen Biotech^1^, was used during RNA extraction by following the kit procedures.

### Cloning of GhGLK1 for Transformation in *Arabidopsis*

To clone the GhGLK gene, *Bam*HI, and *Sac*I, restriction enzymes with forward primer “GAGAACACGGGGGACTCTAGAATG CTAGCTGTGTCACCTTTGAGG” and reverse primer “AC GGGGGACTCTAGAGGATCCCCTCAAAGGTGACACAGCT AGCAT” were used. The vector used in the construction of overexpression recombinant was PBI121, and the specific method of constructing recombinant carrier using the frozen-melt method to transform recombinant plasmids into *Agrobacterium* GV3101 receptor, coated on lysogeny broth (LB) media containing Kanamycin and Rifamycin. After shaking culture for 48 h, a single bacterial colony was picked for PCR and gel electrophoresis confirmations. Floral dipping is used to apply the suspension solution in WT *Arabidopsis* (Lu et al., 2019).

### Validation of GhGLK1 in Drought and Cold Stress Tolerance of Cotton by Virus-Induced Gene Silencing

Using cDNA as a template, the target gene is amplified according to specific primers (forward: GTGAGTAAGGTTA CCGAATTCAGTGAAGGTGGATTGGACGC and reverse: CGTGAGC GGTACCGGATAGCCCCCCCCGCATATGATTGC TCTG). The VIGS vector construction was made using the double enzyme cutting method of *Eco*RI and *Bam*HI enzymes (Yang et al., 2019). The required cultures are TRV2:GhGLK1, empty TRV2:00, and TRV2: PDS. The *Agrobacterium* solution OD600 concentration is approximately 1.5. After injecting cotton leaves, the plants were placed in the dark for 24 h, and later, plants were exposed to normal growth conditions (Mehari et al., 2021). After gene silencing, plants were grown to three leaves for treatment, using a nutrient solution containing 17% PEG6000 to simulate drought treatment, and for cold treatment, 4°C growth chamber temperature at 16 h/8 h light/dark conditions.

### Morphological, Physiological, and Biochemical Trait Determination

Physiological index determination was taken for dehydration rate [excised leaf water loss (ELWL)], relative moisture content [relative leaf water content (RLWL)], ion permeability [cell membrane stability (CMS)], and chlorophyll content [soil plant analysis development (SPAD)] at 0 and 24 h time points. Biochemical index evaluation, mainly SOD, PRO, hydrogen peroxide (H_2_O_2_), and MDA leaf samples, was performed at 0 and 24 h after treatment, and the Solarbio (Solarbio, Beijing, China) test kit was used to determine the enzyme assay according to specific procedures shown in the instructions.

### Cell Damage Identification

Trypan blue and 3,3′-diaminobenzidine (DAB) experiment was performed to see the cell damage of control and treated plants. Taking the leaves of WT and overexpressed *Arabidopsis* plants, before and after drought and cold stress, soaking them in trypan blue dye solution (10 ml of lactic acid, 10 ml of glycerin, 10 g of phenol, 1 0 mg of trypan blue, and 10 ml of distilled water), placing in boiling water bath for 2 min, and later cooling in hydrated chloral (2.5 g/ml), daily replacement of decolorization solution was performed. At least three biological replications were made for each group of three leaf blades. The DAB dyeing experiment used a DAB color rendering kit from the Nanjing Institute of Bioengineering. DAB color rendering work liquid preparation includes reagent A and reagent B in the 1:19 ratio according to the use of mixing and ready to use. Color rendering time is 8–12 h for WT and overexpression *Arabidopsis* leaves in the prepared DAB color rendering liquid. During this time, observe staining to prevent excessive staining. Use alcohol for gradient discoloration. Wash off the blade color 2–3 times with ethanol for 30 min at a time. Use another 70% ethanol to continue to wash off the leaf color 2–3 times for 30 min at a time. Each group of three blades is repeated three times (Li et al., 2019).

### Subcellular Localization of GhGLK1

Using vector pCAMBIA2300-eGFP-Flag, the sequence of GhGLK1 was amplified by forward primer (GAGAACA CGGGGGACTCTAGAATGCTAGCTGTGTCACCTTTGAGG) and reverse primer (ACCCATGTTAATTAAGGATCCACCCAT TGTGGGTGGAAGCC) restriction enzymes containing *Xba1* and *BamH1* cutting sites. Then, the agroinfiltration approach was used to transform the generated recombinant gene into tobacco leaves. Finally, to determine the location of the candidate gene, the transformed tobacco leaves were incubated at 25°C for 24 h in the dark and viewed using fluorescence microscopy (Mehari et al., 2021).

### RT-qPCR Analysis

Leaf samples were taken at 0 and 24 h for the extraction of RNA. RNA was extracted by using the Tiangen Biotech (see text footnote 1) kit. The *GhActin* gene forward sequence ATCCTCTCTTGACCTTG and the reverse sequence TGTCCGCAGGCAACTACTCAT were used as a reference standard for qRT-PCR analysis. For RT-qPCR primer design, the NCBI^2^ website was used. RT-qPCR analysis using AceQ Universal SYBR Green qPCR Master Mix (Vazyme, Nanjing, China) is performed, as detailed in the instruction manual. Specific primers were designed, and RT-qPCR analysis was also performed in four stress-responsive genes, namely, COR15A, RD29A, KIN1, and COR47, to determine their expression in plants after stress treatment (Supplementary Table 1).

### Statistical Analysis

The statistical analysis was performed *via* the SPSS package at the 5% probability level, and when the significant variation was observed, the mean comparison was performed using the Duncan multiple range test (DMRT). The error bars in each graph represent the mean values and SE of three replicates. GraphPad Prism was used to do the graphs of this study. In this study, all the experiments were carried out in three replications.

## Results

### Screening of Overexpressing Lines in *Arabidopsis thaliana*

**FIGURE 1:**
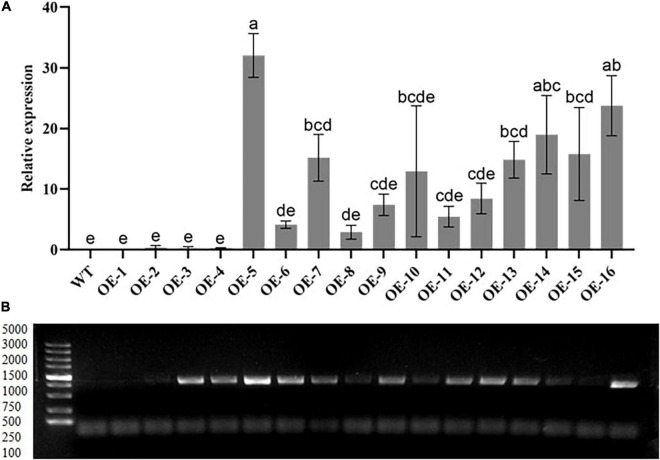
Relative expression levels of transgenic lines by real-time quantitative PCR (RT-qPCR) and gel imaging. **(A)** The transcript expression levels of T2 overexpressing line analysis were performed *via* RT-qPCR. **(B)** PCR investigation performed to check the 783 bp coding sequence in the T2 selection stage using the 5,000 bp marker. OE-1–OE-16 transgenic lines. Three biological replications were taken for each experiment. Means with different letters show significant difference.

Based on the RT-qPCR results of the T2 generation, we selected 3 out of the 16 overexpressing lines with higher levels of GhGLK1 expression. In this study, we selected OE-5, OE-14, and OE-16 for further study (Figure 1). Further screening of T3 generations of expression of pure seeds for phenotype identification and functional verification of the target gene was performed under drought and cold stress.

### Overexpression for Cold and Drought Stress Tolerance

**FIGURE 2:**
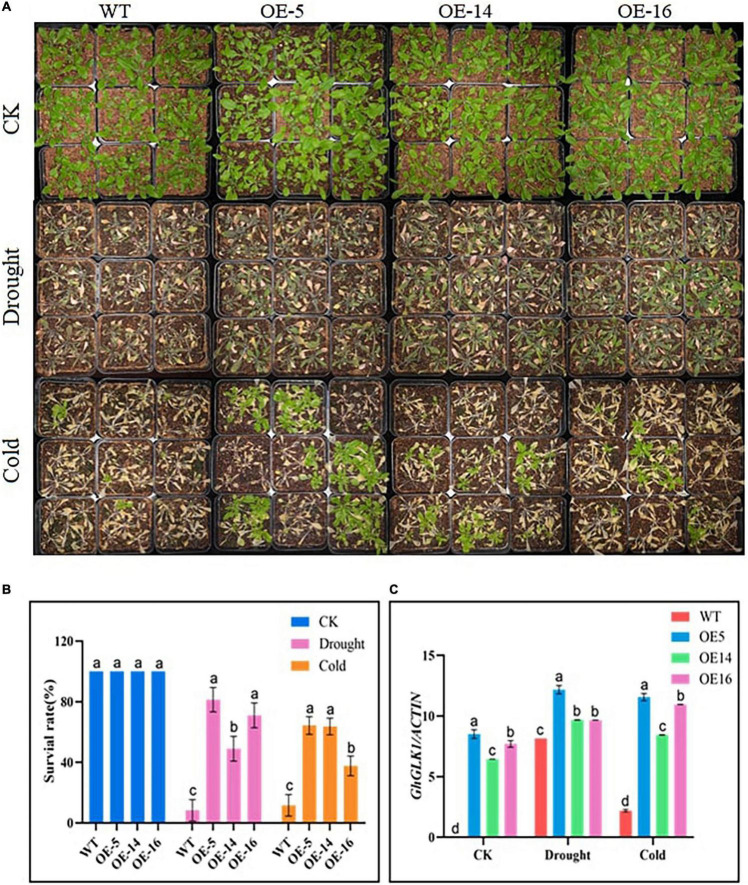
Phenotypic performance of overexpressing lines under drought and cold stress, **(A)** GhGLK1 overexpressing lines and wild type (WT) phenotype in drought and cold stress, **(B)** survival rates of GhGLK1 transgenic lines and WT, **(C)** expression levels of GhGLK1 overexpressing lines and WT in stress conditions of cold and drought. Three biological replications were maintained in the experiment, and Student’s *t*-test was used to determine the mean comparison with ± SD at *p* < 0.05. Means with different letters show significant difference.

We selected 3–4-week-old overexpression lines and WTs with consistent growth in nutritional pots for drought and cold tolerance treatment. In the control group, the phenotype of WT and the three overexpressing lines (i.e., OE-5, OE-14, and OE-16) was the same, but after 20% PEG-6000 treatment for 48 h, the overexpressing lines showed a more resistant phenotype with less wilting symptoms as compared with WT. Whereas, under cold stress, after 7 days of culture at −15°C, the three overexpressing lines somewhat reverted to green, with new leaves, whereas the WT of *Arabidopsis* almost completely withered and turned yellow (Figure 2A). After drought treatment, the survival rates of the three overexpressing lines (i.e., OE-5, OE-14, and OE-16) were 81.3, 49, and 71%, respectively, compared with 8.3% for WT *Arabidopsis*. Under cold stress, the survival rates of the three overexpressing lines (i.e., OE-5, OE-14, and OE-16) reached 64.3, 63.7, and 37.7%, respectively, and for WT 11.7%, the survival rate was recorded. RT-qPCR was used to find the expression level of the GhGLK1 gene, and it was found to be meaningfully higher in three overexpressing lines relative to WT, with the highest expression in the OE-5 line (Figures 2B,C).

### Germination Rate and Root Length Determination

**FIGURE 3:**
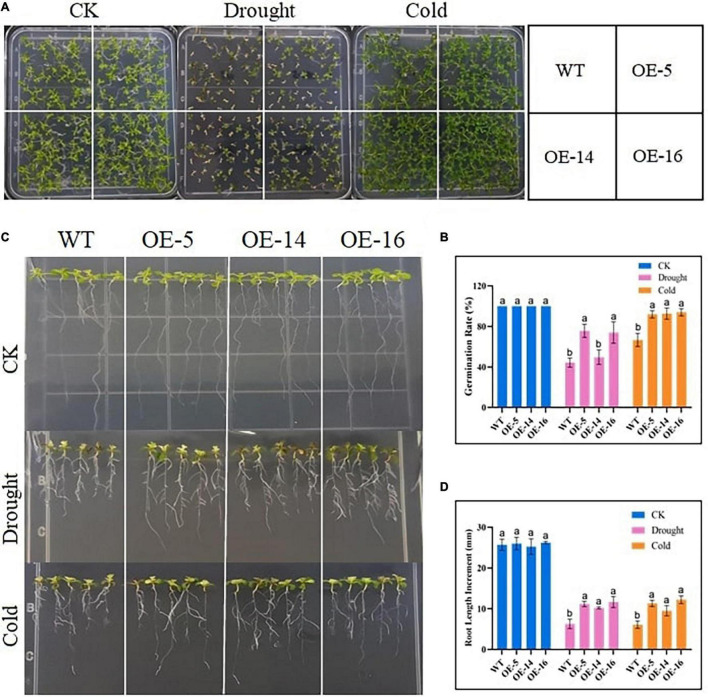
Determination of germination percentage and root length of GhGLK1 overexpressing lines and WT under drought and cold stress. **(A)** Germination percentage of WT and overexpressing lines grown in the control, drought (200 mM mannitol), and cold (4°C); **(B)** germination percentage statistics chart; **(C)** comparison of root growth in the control, drought (200 mM mannitol), and cold (4°C) for 7 days in the WT and transgenic lines; **(D)** determination of root elongation. Three biological replicates for each experiment, and Student’s *t*-test was used to determine the mean comparison with ± SD at *p* < 0.05. WT, wildtype; OE-5, overexpressing line-5; OE-14, over expressing line-14; and OE-16, overexpressing line-16. Means with different letters show significant difference.

Under normal conditions, the germination rate of WT, OE-5, OE-14, and OE-16 overexpression seeds was similar, while the germination rate of the WT after drought stress treatment was only 44.4%, as compared with 75.7, 49.7, and 74% for the three overexpressed lines, and there was a significant difference among OE-5, OE-16, and WT (Figure 3A). The germination percentage of the three overexpressing lines was significantly higher than that of WT germination, indicating that the resistance of seeds to drought and cold increased after the expression of the gene GhGLK1 in the *A. thaliana* (Figure 3B). Root length determination experiments in WT and OE-5, OE-14, and OE-16 lines before treatment were not significantly different, but after drought and cold treatment, the root length of the three overexpressing lines grows meaningfully faster than the WT (Figure 3C). Under normal conditions, the root length of WT and overexpressing lines varies between 25.2 and 26.2 mm, whereas, after drought and cold stress treatments, the root length in WT was recorded with a maximum average of 6.3 mm, and the overexpressing lines scored between 11.2 and 11.6 mm and 9.5 and 12.2 mm in both drought and cold stress treatments, respectively (Supplementary Table 2). The superior performance of overexpressing lines in germination and root growth showed that the GhGLK1 gene has the potential to increase tolerance to both stresses in the overexpressing lines of *A. thaliana*.

### 3,3′-Diaminobenzidine and Trypan Blue Staining Under Drought and Cold Stress in Overexpressed Lines

**FIGURE 4:**
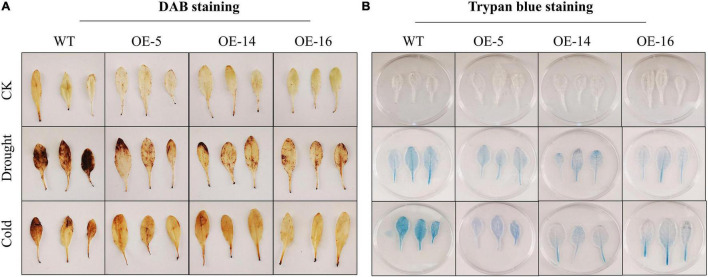
Cell damage evaluation of overexpressing lines and WT under cold and drought stress. **(A)** 3,3′-Diaminobenzidine (DAB) staining; **(B)** trypan blue staining, three biological replicates were taken for each experiment, and Student’s *t*-test was used to determine the mean comparison with ± SD at *p* < 0.05.

3,3′-Diaminobenzidine imaging exhibited that under the normal conditions, the H_2_O_2_ production was very low, and the appearance of brown color was almost invisible. The brown region in the overexpressed *Arabidopsis* leaves was substantially smaller than the WT under drought and cold stress, and the dyeing depth was lighter, and the phenotype was more apparent during drought stress. It is shown that under the same stress, the overexpressing lines suffer less damage (Figure 4A). The results of the trypan blue staining experiment also showed that the area dyed blue in case of overexpressing lines and WT leaves is small under control environments. However, after drought and cold stress treatment, the leaves of overexpressing lines (i.e., OE-5, OE-14, and OE-16) have significantly lesser blue areas than WT leaves. Light blue color indicates that OE-5, OE-14, and OE-16 overexpressing lines suffered less damage than the WT (Figure 4B).

### Measurement of Cell Membrane Stability

**FIGURE 5:**
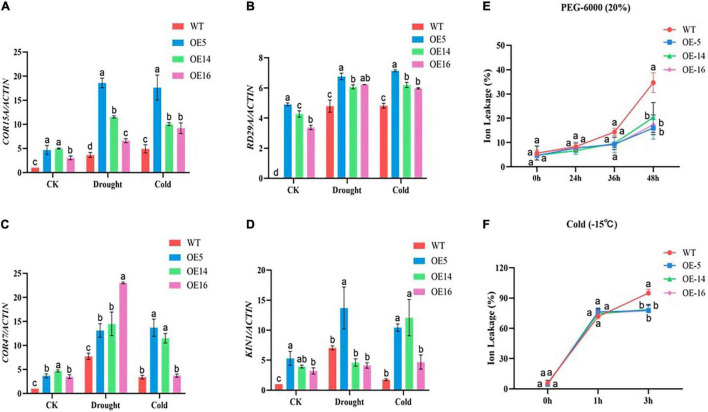
Stress-responsive genes relative expressions and ion leakage determination in GhGLK1 overexpressing lines and WT under drought and cold stress. **(A)** COR15A responsive gene; **(B)** RD29A responsive gene; **(C)** KIN1 responsive gene; **(D)** COR47 responsive gene; **(E)** ion leakage level of GhGLK1 transgenic lines and WT under drought; and **(F)** ion leakage level of GhGLK1 transgenic lines and WT under cold, three biological replicates kept for each experiment, and Student’s *t*-test was used to determine the mean comparison with ± SD at *p* < 0.05. Means with different letters show significant difference.

The ion permeability of WT and OE-5, OE-14, and OE-16 overexpression lines were recorded lower under control situations. In stress conditions of drought and cold, plants experienced injury, and as a result, leaves get damaged, and in this study, we found out that overexpressing lines were more resistant to the said stresses as compared with the WT. The ion exchange in the WT leaves was recorded more relative to the overexpressed lines. With the extension of cold and drought stress for 3 h, the ion permeability of WT kept on increasing but in the case of overexpressed lines, ion permeability was recorded stable. So, we assumed that the cell membrane stability of the overexpressing lines was more as compared with the WT, thus, making overexpressing lines more resistant to drought and cold stress. This suggests that overexpression of the GhGLK1 gene boosted plant resistance to cold and drought stress in the overexpressing lines (Figures 5E,F).

### RT-qPCR Analysis for the Expression of Stress-Responsive Genes

Four stress-responsive genes were investigated in GhGLK to see the relative expression of these genes under both stresses in the wild and overexpressing lines. The results of RT-qPCR revealed that the expression levels of the four stress response genes (i.e., COR15A, RD29A, KIN1, and COR47) were significantly higher in the three overexpressing lines, i.e., OE-5, OE-14, and OE-16, than the WT (Figures 5A–D).

### Subcellular Localization of GhGLK1

**FIGURE 6:**
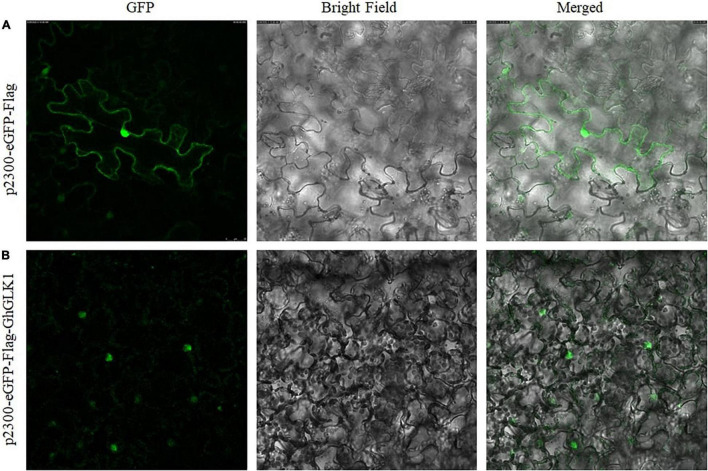
Subcellular localization of pCAMBIA2300-eGFP-Flag-GhGLK1 fusion protein. **(A)** Localization of P2300-eGFP in tobacco leaf cultured with 35 S: pCAMBIA2300-eGFP in GFP, bright field, and merged images; **(B)** tobacco leave transformed with 35 S: GhGLK1 in GFP, bright field, and merged images, respectively. A fluorescence microscope was used to take the image.

The GhGLK1 gene was localized in the nucleus based on online predictions. To further verify whether GhGLK1 was expressed in the nucleus or not, we constructed the pCAMBIA2300-eGFP-Flag-GhGLK1 fusion expression vector. After transformation to the leaves, we observed the subcellular localization *via* fluorescence microscope and found that the empty vector was localized in the nuclear membrane, while the vector carrying the target gene is expressed in the nucleus (Figure 6B).

### Validation of GhGLK1 *via* Virus-Induced Gene Silencing for Its Potential Role in Cold and Drought Stress

**FIGURE 7:**
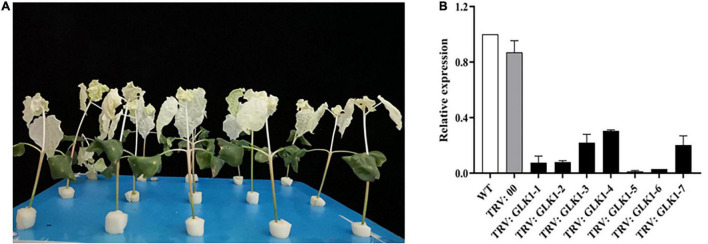
Images for virus-induced gene silencing (VIGS) silencing efficiency and relative expression. **(A)** PDS albino phenotype; **(B)** VIGS interference detection of GhGLK1 gene expression level.

The albino phenotype appeared approximately 8 days after injecting PDS to the cotton leaves, and after 23 days, the albino phenotype was still evident, indicating that gene silencing was successful and the effect was stable (Figure 7A). The expression of GhGLK1 in WT, TRV2:00, and TRV2:GhGLK1 plants was measured using RT-qPCR. No significant variations were observed in the expression of the GhGLK1 gene in WT and TRV2:00, but the expression of the gene in TRV2:GhGLK1 plants was significantly lower than that of WT and TRV2:00, indicating the success of gene silencing (Figure 7B).

### Identification of the Tolerance of Cotton in Drought and Cold Stress Treatments

**FIGURE 8:**
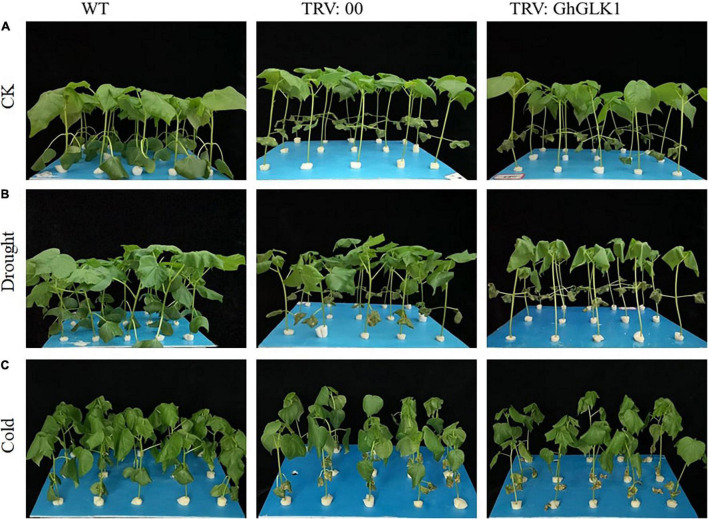
Evaluation of stress tolerance phenotype after silencing GhGLK1 gene. **(A)** Phenotype representation of WT, empty vector, and GhGLK1 under normal condition; **(B)** phenotype representation of WT, empty vector, and GhGLK1 under drought stress; **(C)** phenotype representation of WT, empty vector, and GhGLK1 under cold stress.

In the WT group, growth was more robust, while plants with TRV2:00, TRV2:GhGLK1 suffered mechanical damage during VIGS injection (Figure 8A). After simulated drought treatment of 24 h, WT and TRV2:00 empty plant leaves showed slight wilting, but TRV2:GhGLK1 seedlings were significantly more vulnerable to drought as compared with WT and empty vector (Figure 8B). Higher water loss and wilting in the silenced plants indicate that the drought resistance of the plant was reduced after GhGLK1 silencing. After cold stress treatment of 24 h, there was no considerable difference among WT and TRV2:00 empty vector, but the plants with silent GhGLK1 gene experienced a severe damage than the WT and the empty vector. The stalks of silenced plants softened, and the plants began to show slight signs of inverting, indicating that the cooling resistance of the plant decreased after the gene was silent (Figure 8C).

### Physiological Index Determination of Cotton Under Drought and Cold Stress Conditions

**FIGURE 9:**
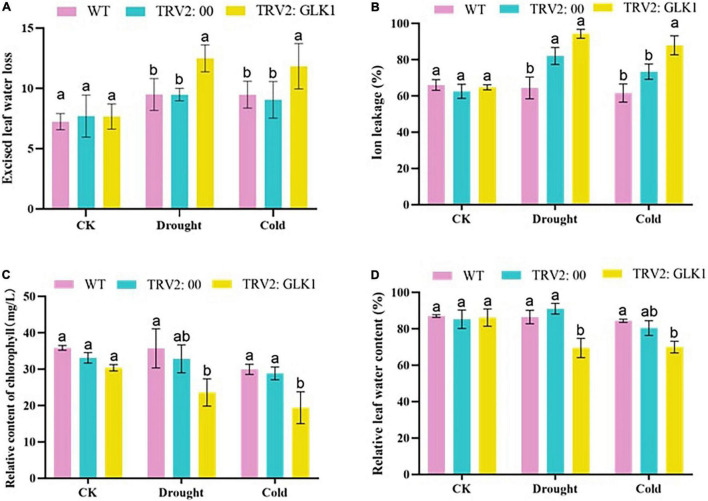
Physiological traits measured in cotton seedlings. **(A)** Excised leaf water loss determination; **(B)** ion leakage level determination; **(C)** determination of chlorophyll content; and **(D)** relative leaf water content determination. Three biological replicates were kept for each experiment, and Student’s *t*-test was used to determine the mean comparison with ± SD at *p* < 0.05. Means with different letters show significant difference.

Evaluation of the water loss percentage in cotton leaves showed that there was no significant variation among WT, TRV2:00, and TRV2:GhGLK1 plants under normal conditions, but after drought and cold stress treatment, the water loss rate in TRV2:GhGLK1 plants was significantly higher than WT and empty vector, indicating a decrease in the water holding capacity of plants after the silencing of the candidate gene. When plants are under stress, damage to the cell membrane causes ions to seep out of the cell membrane. The ion permeability increased significantly in TRV2:GhGLK1 plants than WT and empty vectors, indicating that cell membrane damage was more severe in TRV2:GhGLK1 plants after drought and cold stress. SPAD meter can instantly measure the relative content of chlorophyll in cotton leaves, and the leaf chlorophyll content can indicate the growth of cotton to a certain extent. We observed that under drought stress, the chlorophyll contents of the silenced plants were reduced more as compared with the WT and empty vector plants. After drought and cold stress, the relative water content of silenced plants decreased significantly, compared with WT and plants injected with empty vector (Figure 9). Current results support a decrease in drought and cold resistance of plants after silencing of GhGLK1.

### Determination of Biochemical Indices of Cotton Under Drought and Cold Stress Conditions

**FIGURE 10:**
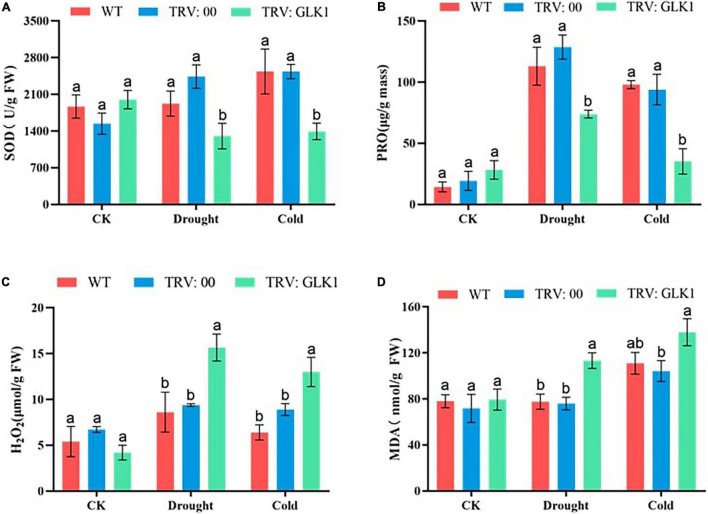
Determination of oxidant and antioxidant enzymes in cotton. **(A)** Determination of superoxide dismutase (SOD) activities; **(B)** determination of proline content; **(C)** hydrogen peroxide (H_2_O_2_) content determination, and **(D)** MDA content determination. Three biological replicates were taken for each experiment, and Student’s *t*-test was used to determine the mean comparison with ± SD at *p* < 0.05. Means with different letters show significant difference.

The SOD contents of plants with the silenced gene were significantly lower than WT and empty vectors under both drought and cold stress conditions. After applying cold and drought stress, the proline content of WT, TRV2:00, and TRV2:GhGLK1 plants increased, but it was significantly lower in TRV2:GhGLK1 gene silent plants than in WT and empty vector plants. After treatment, the H_2_O_2_ content of WT, TRV2:00, and TRV2:GhGLK1 plants increased as compared with control, but it can be seen that the content of H_2_O_2_ in plants with silent gene was significantly higher than WT and empty vector. After stress, MDA levels were significantly higher in plants with TRV2:GhGLK1 silent gene than WT and empty vector, indicating that lipid oxidation was more severe (Figure 10). These results further indicate that plants after silencing the GhGLK1 gene suffer more severe damage and are less resistant to drought and cold stress.

## Discussion

Cold stress, high temperatures, drought, waterlogging, salinity, metal toxicity, and UV radiations are all abiotic stresses that harm plant growth and development, ensuing in major crop losses worldwide (Raj and Singh, 2012; He et al., 2018). Subsequently, plant biotechnology projects should prioritize breeding of crops that are resistant to environmental stresses and forest trees, using molecular regulatory approaches to abiotic stress tolerance which are based on the initiation and control of certain stress-linked genes (Wang et al., 2003). The GARP family of Myb TFs includes GLK TFs, which was linked to chloroplast formation in plants (Chen et al., 2016). GLK genes have been discovered and recognized in various higher plants, and studies have found that they play a crucial role in plant resistance to several abiotic and biotic stresses (Ali et al., 2020). In this study, the overexpression of the GhGLK1 gene in *A. thaliana* and VIGS in cotton was performed to validate the function of this gene. VIGS was used to silence genes after transcription (Lu et al., 2003), and the target gene function was lost or expression level decreased (Purkayastha and Dasgupta, 2009). Silencing LeA4 in tomatoes by VIGS reduces the ability to regulate cell penetration and the survival rate of plants under drought stress, and the accumulation of reactive oxygen species in plants increases (Senthil-Kumar et al., 2008).

In this study, a 783 and 233 bp target gene fragment was inserted into the PBI121 and TRV vectors for overexpression and VIGS, respectively. Through RT-qPCR analysis, the three best lines were chosen in T2 for T3 generation in overexpression of *A. thaliana*. In VIGS, it was found that the gene silencing efficiency reached 85%. In comparison with WT and empty vector plants, plants with silenced GhGLK1 gene showed a substantial decrease in tolerance to drought and cold stress Moreover, excessive water loss and ion leakage, poor chlorophyll, and relative water contents were measured after gene silencing. Similar results were reported by the knockdown of the NAC gene in cotton (Mehari et al., 2021). Currently, the overexpressing lines outperform the WT plants in terms of survival, germination, and root length capability. A report by Lu et al. (2019) stated that an overexpressed gene of DTX/MATE in *Arabidopsis* improves germination rate, root length, and fresh weight increment in the overexpressing lines than the WT plants.

When plants are subjected to various stresses, they produce excessive reactive oxygen species (ROS), of which SOD is an important ROS scavenger, catalyzing superoxide demutualization and playing an irreplaceable role in biological oxidation systems (Sharma et al., 2012; Wang et al., 2018). H_2_O_2_ is a common reactive oxygen molecule in plants, which can oxidize nucleic acids, proteins, and other biological large molecules, and damage the cell membrane, accelerate the aging and disintegration of cells, and can be catalyzed by CAT and POD degradation (Sharma et al., 2012). Proline is widely present in plants and animals as well as microorganisms, and in adverse circumstances, the proline content increases, partly reflecting resistance (Hayat et al., 2012). MDA is one of the products of lipid decomposition, and oxygen free radicals that act on lipid unsaturated fatty acids will produce lipids, so the detection of MDA levels can detect the oxidation level of lipids (Ayala et al., 2014; Morales and Munné-bosch, 2019). In our study, the accumulation of hydrogen peroxide and MDA levels increased significantly, and the content of SOD and proline decreased in both drought and cold stress treatments in the silenced seedlings. Under drought circumstances, the concentrations of oxidant (MDA and H_2_O_2_) and antioxidant (CAT and POD) enzymes in GhMPK3 silenced, and WT plants were determined. The investigation revealed that antioxidant concentrations were much lower in the silenced plants, whereas oxidant levels increased dramatically when compared with the control plants (Yang et al., 2019; Kirungu et al., 2020; Sadau et al., 2021). Moreover, GLKs confer biological stress resistance in crops. NbGlk1 cooperates with Rx1 gene and facilitates antiviral reaction against potato virus in tobacco (*Nicotiana benthamiana*) (Townsend et al., 2018), AtGLK1 has been discovered to improve cucumber mosaic virus resistance (Han et al., 2016) and in rice, the OsGLK1 gene is important for disease resistance (Jarvis and López-Juez, 2013). AhGLK1b has the potential to provide double tolerance to fungi and bacterial infections and abiotic stress resistance (Ali et al., 2020). Previous reports (Powell et al., 2012) stated that overexpression of GLK increased photosynthesis gene profiling in fruits and chloroplast formation, ensuing in improved carbohydrate and carotenoid in tomatoes.

As a reactive oxygen species, H_2_O_2_ is formed in several biological processes. It is regarded to be a vital signaling enzyme in plants that regulate a wide range of physiological and biochemical activities (Niu and Liao, 2016), but also can cause irreparable tissue damage under stress conditions (Bopitiya et al., 2021). H_2_O_2_ accumulates at extremely low levels under normal conditions with no notable variations between the OE lines and WT. After drought and cold stress treatment, the trypan blue and DAB rates were meaningfully different. The leaf of the overexpressing lines has lesser blue areas than those of the wild plants, and with a lighter color depth, it indicates that the overexpressing lines were less damaged. When seedlings face stress, they all accumulate H_2_O_2_, as evidenced by brown matter. The OE lines generated and stored extra H_2_O_2_ than WT seedlings. Studies in soybean reported that the color deepness of the OE seedlings was much lighter than that of the control seedlings when water was scarce or when treated with 250 mM NaCl (Du et al., 2018). In the freezing stress tolerance study, the WT leaves turned yellow or brown, but all transgenic lines stayed green. H_2_O_2_ buildup and cell death were noticed in the WT leaves but not in the transgenic lines as revealed by DAB and trypan blue staining in *A. thaliana* (Chen et al., 2015). Similarly, in cotton GhMKK1, the DAB staining in the overexpressing lines was much lighter after being treated with NaCl, mannitol, and wounding, showing that the level of H_2_O_2_ in the overexpressing lines was meaningfully lower than in the WT in transgenic *N. benthamiana* (Lu et al., 2013). Study by Lu et al. (2013) from a cotton gene in *A. thaliana* stated that GhWRKY6 increased ROS-associated oxidative injury in the OE lines, implying that it increased ROS-associated oxidative injury (Li et al., 2019).

In the current research result, we found out that GhGLK1 was localized in the nucleus. The AhGLK1b gene was found in the nucleus of the peanut and showed the maximum expression in the leaf (Ali et al., 2020). The expression of COR15A, RD29A, KIN1, and COR47, all stress-responsive genes, was considerably higher in the three overexpressing lines than in the WT. This indicates that the overexpressed gene has the potential to regulate abiotic stress tolerance. A report in stress-responsive genes investigated in transgenic lines of GhMPK3 showed upregulated expression compared with the WT (Sadau et al., 2021). Therefore, current results propose that the GhGLK1 might be involved in regulating the genes that play a key role in drought and cold stress tolerance in cotton.

## Conclusion

In this study, we performed functional validation of the GhGLK1 (Gh_D01G0183) gene *via* transformation in *A. thaliana* and gene knockdown in cotton. The overexpression of GhGLK1 in *A. thaliana* results in higher germination and survival rates in both stresses of drought and cold relative to WT seedlings. In stress conditions of cold and drought, the leaf wilting of GhGLK1 silent plants was more serious. Compared with WT and empty vectors, plants with silent GhGLK1 gene have more serious cell damage, more ROS accumulation, and reduced tolerance to drought and cold stress. The experimental findings indicate that the GhGLK1 gene is the true candidate involved in drought and cold stress tolerance, and deeper molecular and genetic mechanisms must be explored.

## Data Availability Statement

The original contributions presented in the study are included in the article/Supplementary Material, further inquiries can be directed to the corresponding author/s.

## Author Contributions

JL: conceptualization and writing-original draft. YX: methodology and analysis. TM and MU: writing, review, and editing. YH, YW, and RP: formal analysis and investigation. KW, XC, and ZZ: resources and validation. FL: funding and supervision. All authors approved the manuscript.

## Conflict of Interest

The authors declare that the research was conducted in the absence of any commercial or financial relationships that could be construed as a potential conflict of interest.

## Publisher’s Note

All claims expressed in this article are solely those of the authors and do not necessarily represent those of their affiliated organizations, or those of the publisher, the editors and the reviewers. Any product that may be evaluated in this article, or claim that may be made by its manufacturer, is not guaranteed or endorsed by the publisher.

## Footnotes

1
www.tiangen.com

2
https://www.ncbi.nlm.nih.gov/
