# Supplementary material for: GhGLK1 a Key Candidate Gene From GARP Family Enhances Cold and Drought Stress Tolerance in Cotton
Source: Front Plant Sci. 2021 Dec 16;12:759312. doi: 10.3389/fpls.2021.759312 (PMC8725998; doi:10.3389/fpls.2021.759312)
Supplement: Supplementary file 7 [file Data_Sheet_1.docx]

**Supplementary figure captions**

**Supplementary figure 1:** PCR amplification and gel band formation was done to check 783bp coding sequence in the T2 selection stage using 5000bp marker

**Supplementary figure 2:** PCR amplification and gel band formation was done to check the Agrobacterium cloned VIGS gene

**Supplementary table captions**

**Supplementary table 1:** List of primers for cloning, stress responsive genes and RT-qPCR analysis

**Supplementary table 2:** data for germination rate, root length and survival rate of Transgenic and wildtype during drought and cold stress treatment

**Supplementary table 3:** Data for cell membrane stability and relative expression of Transgenic and wildtype during drought and cold stress treatment

**Supplementary table 4:** data for silencing efficiency of wild type, positive control and silenced gene seedlings

**Supplementary table 5:** Physiological traits (ELWL, RLWC, CMS and Chlorophyll content) of the wildtype, positive control and Silenced gene in VIGs experiment

**Supplementary table 6:** Biochemical parameters (SOD, Proline, MDA and H_2_O_2_) of the wildtype, positive control and Silenced gene in VIGs experiment
