# Supplementary figures and images for: GhGLK1 a Key Candidate Gene From GARP Family Enhances Cold and Drought Stress Tolerance in Cotton
Source: Front Plant Sci. 2021 Dec 16;12:759312. doi: 10.3389/fpls.2021.759312 (PMC8725998; doi:10.3389/fpls.2021.759312)

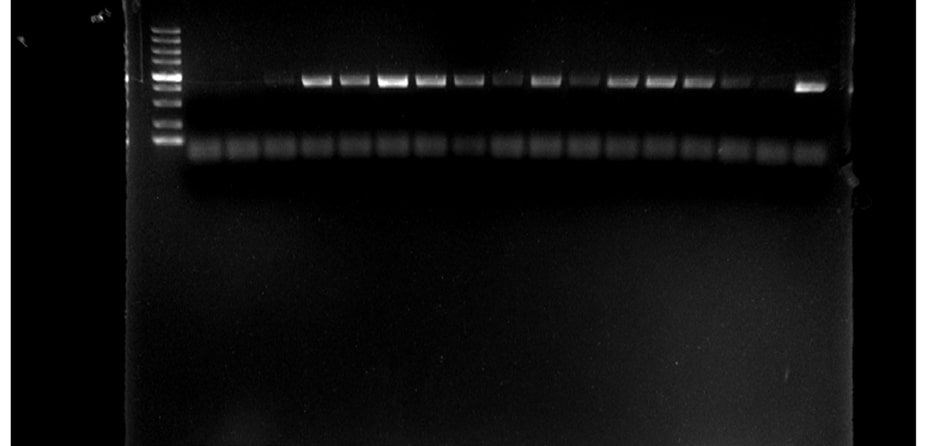

Supplement: Supplementary file 8 [file Image_1.JPEG]

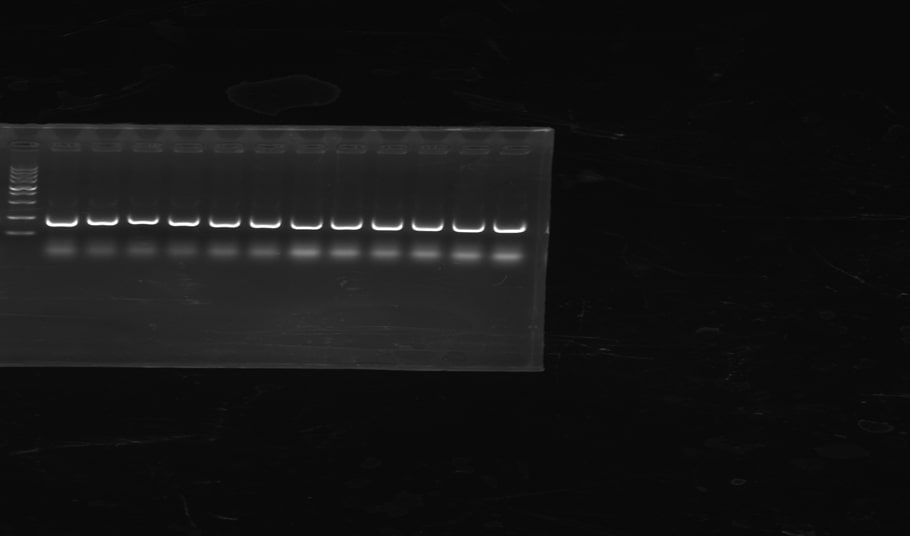

Supplement: Supplementary file 9 [file Image_2.JPEG]
